# Differential requirement for RecFOR pathway components in *Thermus thermophilus*

**DOI:** 10.1111/1758-2229.13269

**Published:** 2024-06-01

**Authors:** Cristina L. Gómez‐Campo, Ali Abdelmoteleb, Verónica Pulido, Marc Gost, Dione L. Sánchez‐Hevia, José Berenguer, Mario Mencía

**Affiliations:** ^1^ Center for Plant Biotechnology and Genomics (CBGP) Polytechnic University of Madrid Madrid Spain; ^2^ Department of Molecular Biology Science Faculty, Center for Molecular Biology Severo Ochoa (CBM), Autonomous University of Madrid‐Higher Council of Scientific Research (CSIC) Madrid Spain; ^3^ Department of Botany, Faculty of Agriculture Menoufia University Shebin El‐Kom Egypt; ^4^ Department of Medical Biochemistry and Microbiology Uppsala University Uppsala Sweden

## Abstract

Recombinational repair is an important mechanism that allows DNA replication to overcome damaged templates, so the DNA is duplicated timely and correctly. The RecFOR pathway is one of the common ways to load RecA, while the RuvABC complex operates in the resolution of DNA intermediates. We have generated deletions of *recO*, *recR* and *ruvB* genes in *Thermus thermophilus*, while a *recF* null mutant could not be obtained. The *recO* deletion was in all cases accompanied by spontaneous loss of function mutations in *addA* or *addB* genes, which encode a helicase‐exonuclease also key for recombination. The mutants were moderately affected in viability and chromosome segregation. When we generated these mutations in a Δ*ppol/addAB* strain, we observed that the transformation efficiency was maintained at the typical level of Δ*ppol/addAB*, which is 100‐fold higher than that of the wild type. Most mutants showed increased filamentation phenotypes, especially *ruvB*, which also had DNA repair defects. These results suggest that in *T. thermophilus* (i) the components of the RecFOR pathway have differential roles, (ii) there is an epistatic relationship of the AddAB complex over the RecFOR pathway and (iii) that neither of the two pathways or their combination is strictly required for viability although they are necessary for normal DNA repair and chromosome segregation.

## Graphical abstract

In *Thermus thermophilus*, disrupting the highly conserved *recFOR* pathway, essential for bacterial recombinational repair, along with the *ruvB* helicase, reveals that *RecF*, *RecO* and *RecR* have distinct roles. This contrasts with their functions in other bacteria.

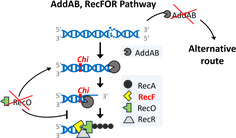

## INTRODUCTION

To be able to use and transmit their genetic information, organisms must ensure that their genetic material is not severed or deteriorated. In bacteria, recombinational repair of chromosome lesions is one of the main processes responsible for the integrity of the DNA (Kuzminov, 1999, 2011). Two pathways have been known to be key in homologous recombination, the RecBCD/AddAB pathway (AddAB for short, hereafter) that acts on double‐strand breaks (DSB), and the RecFOR pathway, dedicated to single strand gaps (SSG) (Michel & Leach, 2012; Spies & Kowalczykowski, 2014; Yeeles & Dillingham, 2010). Both pathways have been shown to be not only key for the repair of the genome, but also for the incorporation of environmental DNA (eDNA) promoting events of horizontal gene transfer (HGT) (Ithurbide et al., 2020; Wang et al., 2011). The AddAB complex has helicase and nuclease activities that proceed degrading the dsDNA ends until *chi* sequences present in the corresponding bacterial genomes are detected. Then, the AddAB complex starts to generate 3′ ssDNA overhangs, which will be first covered by SSB protein, and this, in turn, will be eventually displaced by the RecA filament (Yeeles & Dillingham, 2010). The RecFOR proteins bind to ssDNA gaps or regions with 3′ ssDNA extensions and modulate the displacement of SSB proteins and promote polymerization of RecA on the SSB‐complexed ssDNA (Lenhart et al., 2014; Morimatsu et al., 2012). So, both pathways converge in recruiting RecA to ssDNA, which will lead to the homology‐guided strand exchange between the damaged and the intact sister DNA molecules, central to recombinational repair (Prentiss et al., 2015).

The proteins encoded by the *recFOR* genes have long been considered to form a complex (Honda et al., 2006), or otherwise work in concert, although this view is changing (Sakai & Cox, 2009; see also Henry et al., 2023). In *Escherichia coli*, it has been recently reported that RecF and RecO do not normally colocalize and they seem to play distinct functions time‐spatially separated (Henrikus et al., 2019). In line with this, it has been observed that RecF is not present in some species, while RecOR genes are widespread (Rocha et al., 2005). Additionally, in *Bacillus subtilis*, RecOR is not only required in the SSG repair but also downstream the AddAB route, to displace the SSB and load RecA onto the ssDNA (Lenhart et al., 2014). In contrast, *Helicobacter pylori* RecOR is involved in intragenomic recombination, rather than in DSB repair (Wang et al., 2011). Regarding *Deinococcus radiodurans*, the deletion of the *recFOR* genes produces a phenotype similar to that observed upon *recA* deletion, with clearly impaired growth and radiosensitivity (Bentchikou et al., 2010), and, besides, in this bacterium the RecFOR proteins are also involved in transformation (Ithurbide et al., 2020). In several species studied, the simultaneous elimination of the RecFOR and AddAB pathways leads to cells with severe defects in recombinational repair (Carrasco et al., 2015; Marsin et al., 2010; Zuñiga‐Castillo et al., 2004). In any case, it appears that, in bacteria, the roles of the different recombination factors and their integration in the main recombination routes are complex and follow group‐specific patterns.

After RecA action, a Holliday junction (HJ) is commonly formed and, in *E. coli* and other species, this structure is usually processed by the RuvABC complex to yield two separate DNA molecules (West, 1997). To do this, the HJ is first recognized by RuvA, then the helicase activity of RuvB catalyses the branch migration of the structure (Yamada et al., 2002) and, finally, the resolvase RuvC cuts at two positions of opposite polarity in the HJ to produce the resolution of the structure. As an additional related role, RuvAB has also been shown to be able to catalyse the replication fork reversal when the replication machinery is stalled (Baharoglu et al., 2006).

The *Deinococcus‐Thermus* group is an ancient phylum of bacteria that contains the extreme thermophilic *Thermus* genus and the radioresistant *D. radiodurans* species among others (Ho et al., 2016). Adaptation of these bacteria to extreme environments has involved characteristic gene gains and losses. In *Thermus* for instance, genes traceable to thermophilic archaea are present, while in *Deinococcus*, radiation‐resistance genes acquired from other bacteria are more common (Omelchenko et al., 2005). *Thermus thermophilus* (Tth) is the best characterized species of the genus (Cava et al., 2009), and it has served as a source of thermophilic enzymes. Together with that, it must be emphasized that an important effort has been directed to the structural elucidation of its proteome (Yokoyama et al., 2000), to the point where the 3D structure has been determined for up to 25% of the proteins encoded by its genome (Jenney Jr & Adams, 2008). For example, the structures of Tth RecF, O and R, alone or, in the case of RecO, in complex with DNA (Aono et al., 2003; Chaudhary et al., 2020), have been described, and, very recently, the structure of Tth RecF, O, and R in complex with a dsDNA end has also been reported, which has allowed to understand the network of interactions of these proteins when working in concert. Also the structures of RuvB and RuvC, this again with DNA (Chen et al., 2013; Górecka et al., 2013; Yamada et al., 2001) have been described. The *addAB* genes have been identified in Tth (Gurung & Blumenthal, 2020) although their structures have not been solved so far.

Natural competence is a very interesting and useful characteristic of the genus *Thermus*, being in general, constitutive, and showing different efficiencies across strains. Notwithstanding, while the transformation machinery has been described (Blesa et al., 2018; Schwarzenlander & Averhoff, 2006), there are still many unknown aspects about this process in Tth. At the same time, this DNA acquisition ability has probably prompted the incorporation of a panoply of defence mechanisms in *Thermus*, such as several CRISPR systems (Artamanova et al., 2020) or the Argonaute protein (Swarts et al., 2014) to filter out the possible negative effects of eDNA. Another important characteristic of this species is the polyploidy of its genome, a fact that probably has a clear impact on the evolution of recombinational repair processes.

Despite all the available structural information, the way DNA recombinational repair routes operate in vivo in this organism has received little attention. In this article, we have generated deletions of the *recO*, *recR* and *ruvB* genes and studied their effects on viability, DNA damage resistance, and transformation efficiency. The mutants were verified by whole genome sequencing, and we observed that *recO* mutant strains had probably acquired compensatory mutations in the form of frameshifts in the *addA* or *addB* genes and, in some cases, point mutations in the *recA* gene. In view of this, we repeated the analysis on a markerless primase polymerase (*ppol*) and *addAB* locus region deletion background (*ppol/addAB*) (Verdú et al., 2022). The results show that this background alleviates some of the defects produced by RecO, RecR or RuvB elimination, but exacerbates others. Given that double mutants are viable and show a greatly increased transformation efficiency, additional route/s of recombinational repair must be active in Tth to support viability and transformation in the absence of the AddAB and RecFOR pathways.

## RESULTS

### Mutant strain construction and sequencing

**FIGURE 1:**
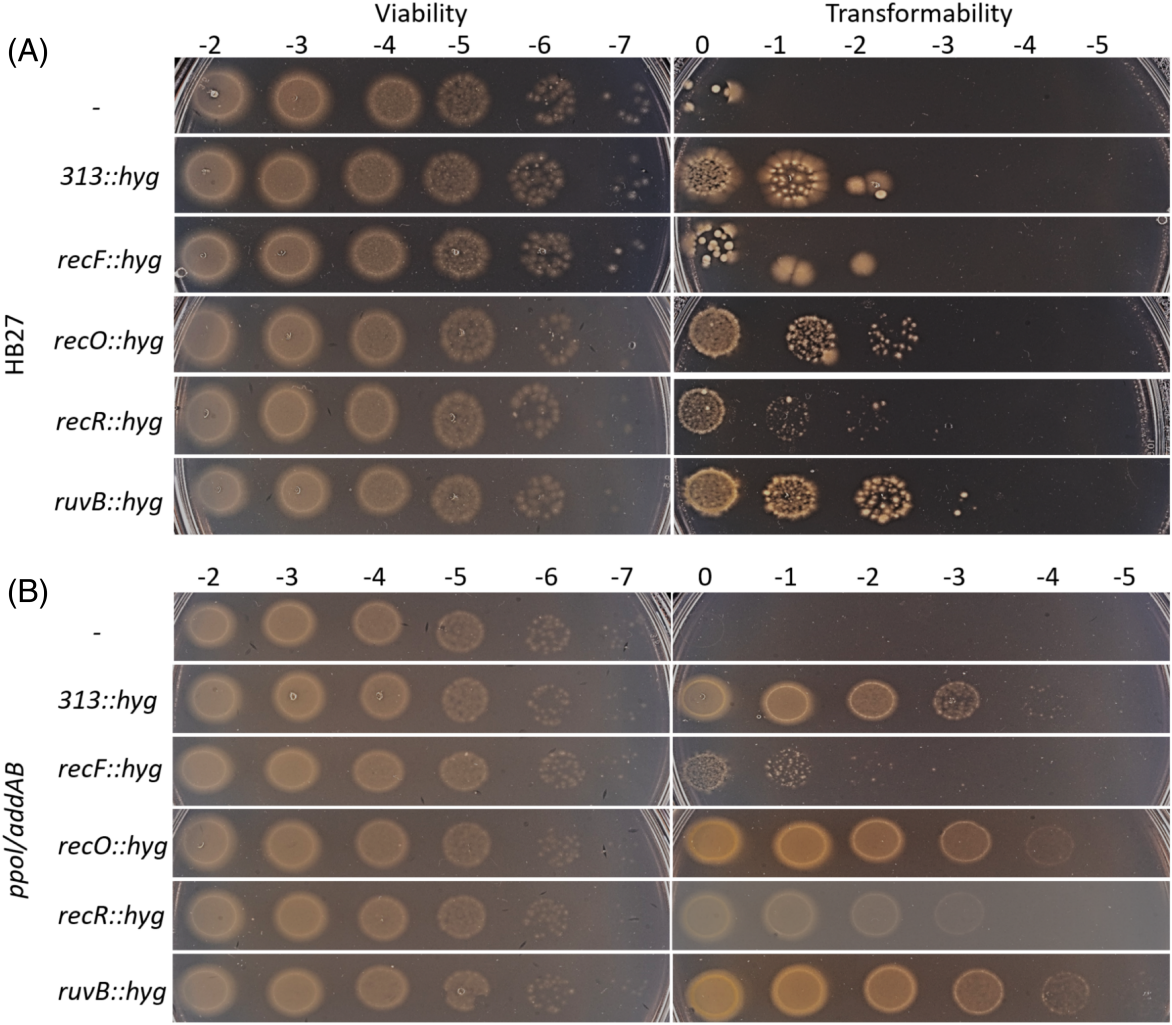
Transformability spot‐assay of *Thermus thermophilus* with DNA constructs to delete the indicated genes. Serial dilutions of transformed cells (Tth HB27 strain, A; or *ppol/addAB* strain, B) with the indicated construct were plated in non‐selective plates (left panels) or plates supplemented with hygromycin (right panels). Cells were spotted on TB agar plates without antibiotic (viability) or with hygromycin and incubated at 65°C for 48 h.

We prepared plasmid constructs to perform the deletion of the *recF*, *O*, *R* and *ruvB* genes (TTC1721, TTC0258, TTC1236 and TTC0038, respectively; details of the constructs are available upon request) by double‐recombination substitution of the target genes by a cassette conferring thermostable hygromycin resistance (*hyg*) in Tth. After transformation of the strain Tth HB27 with lineal forms of the *ruvB* construct, we could obtain a number of colonies (Figure 1A) even higher than that yielded by a similar construct that deleted the non‐essential gene TTC0313 used as control (Alvarez et al., 2014), while the *recO* and *recR* constructs produced also numerous but smaller colonies. However, the *recF* construct produced very few colonies that, upon PCR verification (see Methods section), were shown not to be bona fide *recF* deletion mutants. After two successive re‐streaking of individual colonies, we isolated three mutant strains for each of the mutants and sequenced their genomes to check for the correct insertion of the *hyg* cassette and to search for the presence of putative compensatory mutations in the genome. The strains, with their corresponding genotypes, were named RecO1 to O3, RecR1 to R3 and RuvB1 to B3.

Whole genome sequencing showed that all mutant strains had correctly inserted the *hyg* resistance cassette replacing the corresponding genes. However, several additional mutations were detected across the genome in all the cases. Operatively, we classified these mutations in those for which we could discern a functional relationship with the deleted genes (class 1, Table 1) and those for which we could not (class 2, in Table S1). Regarding the class 1 mutations, the most remarkable fact is that the three *recO* mutants had also acquired a loss of function by frameshift mutation in one of the genes for either *addA* or *addB* (Table 1). As mentioned in Introduction, the AddAB helicase‐nuclease complex is one of the most important players in homologous recombination in many bacteria, and it is thought that RecFOR operates after AddAB has left 3´ssDNA extensions (Lenhart et al., 2014). Additionally, the RecO1 and RecO3 strains bear point missense mutations in the *recA* gene (Ala80Val and Ala80Ser, respectively, Table 1). All this suggests that AddAB needs to be inactivated for *recO* mutants to be viable, as a compensatory mechanism, and that this may be helped by specific *recA* point mutations.

**TABLE 1:** List of genomic mutations for which we can discern a relationship with the deleted gene (class 1). For each mutation position and change, strain in which it is present, gene name, annotated protein, predicted amino acid change and protein total length are shown.

Position	Mutation	HB27 RecO1	HB27 RecO2	HB27 RecO3	Gene name		Change	Protein length
627140	GC_G	1	0	1	TT_C0638	AddA	Frameshift Ala 311	857
629176	GC_G	0	1	0	TT_C0639	AddB	Frameshift Ala 369	737
1390675	G_A	0	0	1	TT_C1466	RecA	Ala80Val	340
1390676	C_T	1	0	0	TT_C1466	RecA	Ala80Ser	340
		**HB27 RuvB1**	**HB27 RuvB3**					
1857615	T_C	1	1		Intergene TT_C0902	RecG	Position 130 upstream ATG	
		**Ppol RuvB4**	**Ppol RuvB5**	**Ppol RuvB6**				
352672	C_T	1	1	1	TT_C0366	Type 4 UDG‐DNA glycosylase	Ala19Thr	205

The *recR* mutants do not appear to have additional class 1 changes typical or exclusive of this series that could be associated to compensatory mechanisms. The mutant strains RuvB1 and RuvB3 carry a T to C substitution 130 bp upstream the ATG of the gene coding for RecG, a double helicase involved in replication fork regression and recombination (Rudolph et al., 2009). This would suggest that the loss of *ruvB* could perhaps be compensated by a mutation on the *recG* promoter.

The additional, class 2 mutations (see Table S1) found, do not show any specific pattern for the deletions performed and, as mentioned, they are not easy to interpret in light of the possible pathways affected by the deletions.

In previous work, we have described a markerless Tth mutant strain (*ppol/addAB*) that has a deletion on the primase‐polymerase gene (*ppol*) and a spontaneous 14 kb deletion affecting 12 genes including the complete loss of *addA* and *addB*. This strain appears to be genomically stable and has a high transformation efficiency (around 1000‐fold over the wild type) (Verdú et al., 2022). Given that *addA* or *addB* mutations were associated to the *recO* deletion, we decided to generate the *recF*, *recO*, *recR* and *ruvB* knockouts on the *ppol/addAB* background to see if some kind of compensation was also observed. These new mutant strains were named RecO4 to O6, RecR4 to R6 and RuvB4 to B6.

Again, all the mutant strains could be obtained with high efficiency of transformation, typical of the *ppol/addAB* background, except for the one carrying the *recF* deletion (Figure 1B). In this case, upon the initial transformation very small colonies appeared, which, after re‐streaking in selective medium, turned out not to be viable. Since this bacterium has been reported to be polyploid, an interpretation of this is that the initial colonies could be heterozygous, keeping copies of wild‐type *recF* together with chromosomes with the *hyg* cassette insertion. Streaking onto new plates would force the expansion of the *hyg* cassette until complete replacement of *recF* which would cause cell death. To confirm the essentiality of *recF*, we expressed hexahistidine‐tagged RecF from a plasmid to try to complement the chromosomal deletion of the *recF* gene (Figure S1A), both in HB27 and *ppol/addAB* backgrounds. A slight toxicity of the RecF expression constuct was observed in *ppol/addAB*. In neither of the strains, was possible to observe complementation of the chromosomal *recF* deletion. The colonies observed in the spots without dilution were checked by PCR and they were not bona fide *recF* mutants. Western blotting with an anti‐his tag antibody showed no band corresponding to RecF (Figure S1B), while it could detect the expression of a non‐relevant Tth protein (gene TTC0803) in the ppol/addAB background. We do not know the reasons for the lack of expression of RecF, especially considering that we have used a tested vector pMoTH3A (Verdú et al., 2019) or the reason for the toxicity of the construct. With this, we cannot determine if *recF* is essential, or, otherwise, that the effect we are observing would be due to the deletion construct disrupting the expression of some other neighbouring gene/s, in turn, essential.

Whole‐genome sequencing showed again that the deletions were the correct ones in each of the *recO*, *recR* and *ruvB* strains and, once subtracting the *ppol/addAB* variant profile, the remaining mutations were much less in number than those observed for the deletions generated on the HB27 wild type background (Table S2). Here, we observed the following class 1 mutations: in RecR6, a mutation causing the change Asp138Gly in RecA; in the three *ruvB* mutant strains an Ala19Thr change in a type 4 uracyl‐DNA glycosilase and a Leu103Pro in the gene TTC_0657, which is immediately downstream of the *ppol* gene. Additionally, there is an Ala72Val in the ribosome binding factor A gene (class 2 mutation). Finally, for both backgrounds, HB27 or *ppol/addAB*, few mutations mapped at the megaplasmid present in the strain, pTT27, and no class 1 mutations were assigned (Table S3).

### The mutants show different viabilities at 65°C

**FIGURE 2:**
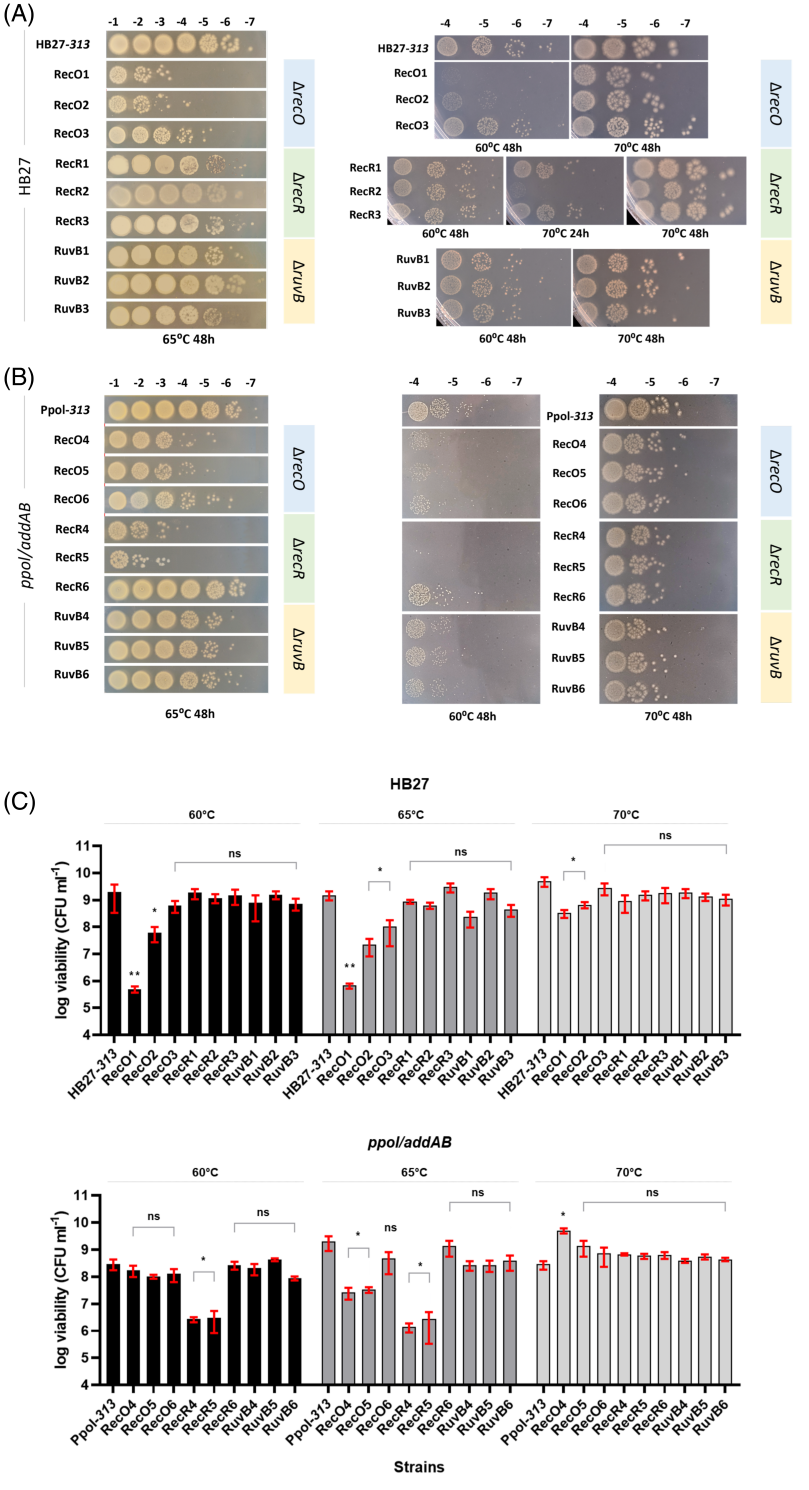
Cell viability and temperature effect on *Thermus thermophilus* mutants. (A) Decimal dilutions of cultures of the HB27‐*313* control or the different mutants as described in the main text, constructed in HB27A genetic background were spot‐plated (10 μL) on non‐selective plates and incubated at the indicated temperatures commonly for 48 h, unless indicated otherwise. (B) As in (A) with mutants constructed in *ppol/addAB* background. (C) Quantification of viability of the strains at growth temperatures shown in (A) and (B). The cells were plated on SC plates without antibiotic. The histograms represent the number of viable cells per mL, with the corresponding SDs. Statistic treatment was performed as indicated in Materials and Methods.

In order to see how the different mutant strains were affected, we performed spot‐plating experiments at different temperatures (Figure 2). We chose 60*°*C as restrictive temperature, 65*°*C as standard growth condition since that is the functional temperature limit for the enzymes that allow antibiotic selection and 70*°*C as optimal growth temperature. In all cases, the plating was done in the absence of antibiotic. At 70*°*C Tth HB27 grows faster than at 65*°*C, and at 60*°*C the growth is visibly impaired.

At 65*°*C, on the HB27 wild type background, we observed that the RecR and RuvB series mutants (Figure 2A) display inferior viabilities, yet within an order of magnitude of that of the wild‐type strain (Figure 2C), and the differences are not significant. On the other hand, the RecO1 and RecO2 mutants show a clearly impaired viability respect to the control: 3 logs lower for RecO1, about 2 logs for RecO2, and 1 log for RecO3. This suggests that *recO* deletion has a clear impact on the survival of Tth, an impact that can be alleviated by compensatory mutations, as the ones detected, and commented above. This compensation appears to be incomplete for RecO1 (with a frameshift in *addA* plus Ala80Ser in RecA), intermediate for RecO2 (frameshift in *addB* and no change in *recA*) and better but not complete for RecO3 (with the same frameshift in *addA* as RecO1, plus Ala80Val in RecA in this case). Apparently, the frameshift in *addA* can potentially couple a higher loss in viability than that of *addB* and, adding on the *addA* mutation, the RecA Ala80Val produces a much better compensation than Ala80Ser. This is, assuming that none of the other mutations observed in this strain are very relevant for the complementation phenotype, which may not be the case. Curiously, the *recR* mutants that appeared to have impaired viability just after transformation of the knockout construct (Figure 1A), now show better viability, also suggesting some kind of compensation.

In the *ppol/addAB* background at 65*°*C (Figure 2B,C), the *ruvB* mutants have around 10‐fold lower viability than the control, the RecO4 and RecO5 around a 100‐fold lower, and the *recR* mutants show a viability impaired by three logs, except for the RecR6 strain (the one with the change D138G in RecA), with a viability just slightly lower than the control. In the case of RecO6, we could not find any mutation that would explain the increased viability.

At 60*°*C, we observed similar results to those obtained at 65*°*C relative to the controls, taking into account that all the strains show slower growth at 60*°*C. Only the defect in viability of RecO3 is not significant in this case in the wild type, and in the *ppol/addAB* background the RecO4 and RecO5 defects are visible but not significant.

At 70*°*C, all the strains grow well and the defects showed for RecO1 and RecO2 have been alleviated, although not completely. At this temperature RecR2, experiences a lag in growth of about 24 h, but eventually reaches a growth comparable to the other *recR* mutants after 48 h of culture. In the *ppol/addAB* background, at 70*°*C, all the mutants behave similarly to the control strain, only RecO4 seems to have a slightly better viability. We do not know the reason for this.

From the previous results, we can say that most mutants have certain defects in viability when cultivated at 60*°*C and 65*°*C although these defects are not statistically significant. Notably, there are significant defects observed, particularly in HB27 RecO1 and RecO2, as well as in the *ppol/addAB* background RecR4 and RecR5. Interestingly, most of the defects seem to diminish or vanish entirely at 70*°*C.

### Sensitivity to DNA damage agents

**FIGURE 3:**
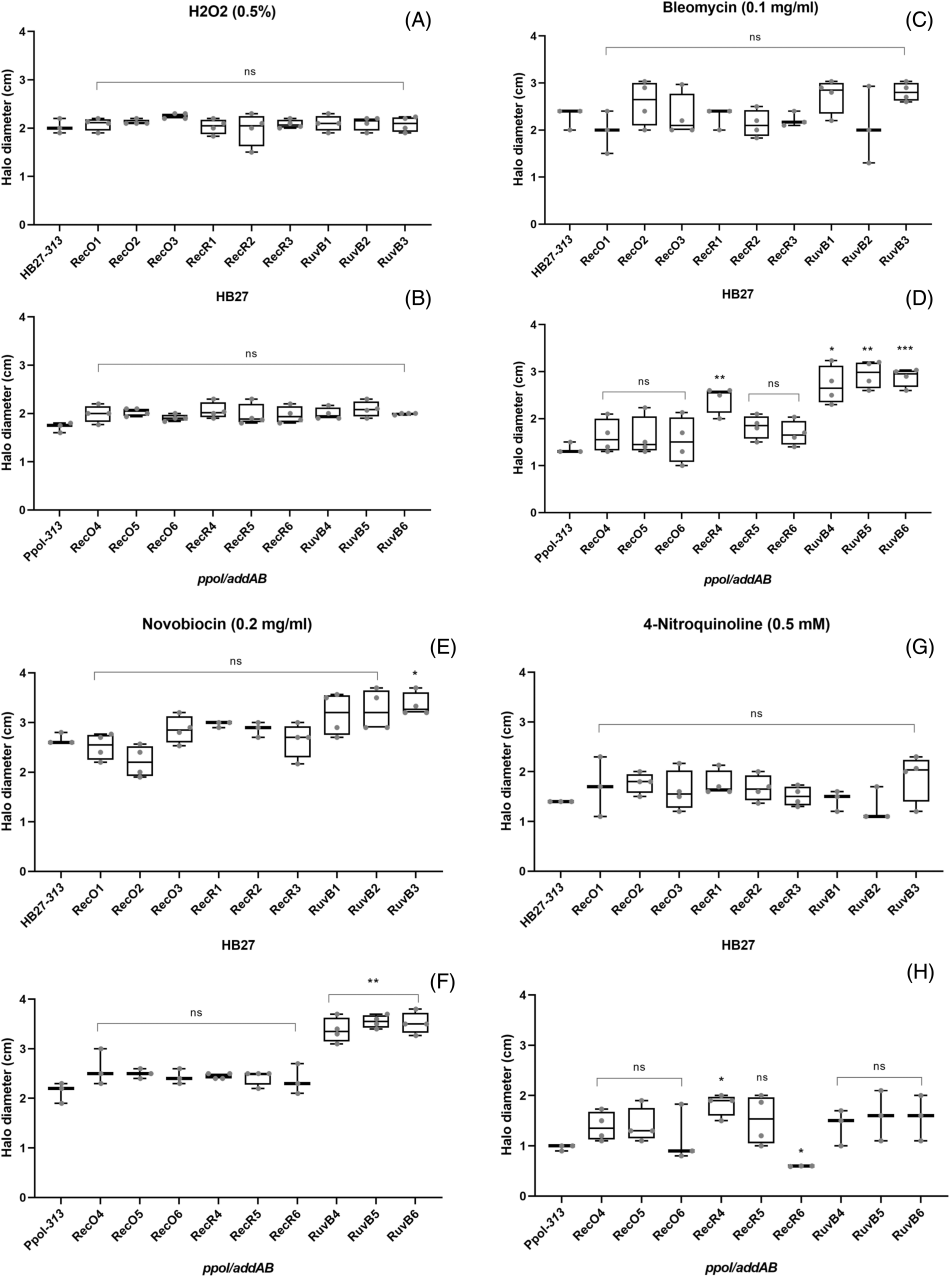
Effect of DNA‐damaging agents on *Thermus thermophilus* mutants. Representation of the inhibition halo diameter, in cm, observed for the control strain or the indicated mutants, in HB27 or in *ppol/addAB* background as indicated. A number of 10^7^ to 10^8^ cells, according to the viability of the strain, of overnight saturated cultures were seeded on TB plates, on top of which 5 mm cellulose disks were placed. The disks were embedded with 10 μL of the different indicated DNA damaging agents: hydrogen peroxide (H_2_O_2_) (0.5%) (A, B), bleomycin (0.1 mg/mL) (C, D), novobiocin (0.2 mg/mL) (E, F) or 4‐Nitroquinoline‐N‐oxide (4‐NQO) (0.5 mM) (G, H). The plates were incubated at 65°C for 48 h. Diameter of halos is the average of at least three biological replicates per mutant strain. Asterisks indicate significant statistical differences (0.12 (ns), 0.033 (*), 0.002 (**), <0.001(***)) between the different mutant strains with the mean of control strains, either in wild type or in *pppol/addAB* background.

Given that most mutant strains have viability defects under standard growth conditions we wanted to test if these viability defects were connected to specific DNA repair pathways. Thus, we performed growth inhibition halo measurements using cellulose disks soaked with compounds that produce different types of DNA damage according to the Kirby‐Bauer method (Figure 3).

Oxidative damage produced by hydrogen peroxide (H_2_O_2_) did not produce significant differences in the diameter of the inhibition halo observed for all series of mutants either in HB27 or *ppol/addAB* background (Figure 3A,B). The bleomycin treatment (causing DSB) (Figure 3C,D) did not raise significant differences in the mutants derived from HB27 relative to the control strain. However, interestingly, the bleomycin produced smaller halos in the *ppol/addAB* background than in the wild type, supporting an unexpected increased resistance to DSB promoted by the *ppol/addAB* deletions. Moreover, this increase in resistance in the *ppol/addAB* background was not significantly changed in the mutants, with the exception of RecR4 and the RuvB series in which the resistance was decreased to levels similar to that showed by HB27 derivatives. With novobiocin (an inhibitor of GyrB) (Figure 3E,F), only RuvB6 was apparently more sensitive in the HB27 background, while in the *ppol/addAB* background, the RuvB series appeared to be significantly more sensitive than the corresponding control strain. Lastly, 4‐NQO was used to mimic UV‐light damage (Williams et al., 2010), which produced a slight increase in sensitivity in all the mutants (Figure 3G,H), but this increase was not statistically significant, except for *ppol/addAB* RecR4 and RecR5. This set of results suggests that the *ruvB* deletion probably impairs recombinational repair of DSBs caused by bleomycin, and probably also those generated by novobiocin treatment.

### The enhanced transformability of the ppol/addAB background is not affected in the rec or the ruvB mutants

**FIGURE 4:**
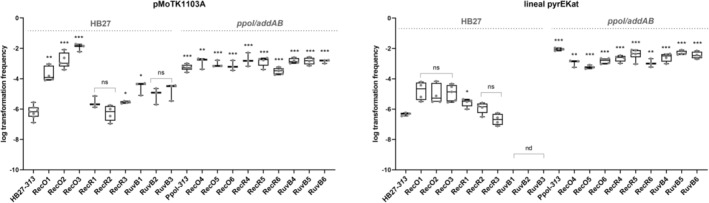
Transformation efficiency of *T. thermophilus* mutants. One hundred nanograms of a replicative plasmid (pMotK1103A) or an integrative linear DNA fragment conferring resistance to Kn (pyrEKat), were used to transform the indicated strains. Serial dilutions of 500 μL cultures of transformed cells were plated on Kn/Hyg and Kn plates and incubated for 48 h at 65°C. The transformation efficiency is equal to the number of colonies on Kn/Hyg plates, relative to the corresponding number of viable colonies on Kn plates. Transformation frequencies are represented in a box and whisker plot with at least 3 biological replicates per mutant strain (individual values, blue dots). Asterisks indicate significant statistical differences (0.12 (ns), 0.033 (*), 0.002 (**), <0.001(***)) between the different mutant strains with the mean of control strains, either in wild type or in *pppol/addAB* background.

Next, we assayed the effect of the different mutant series on the ability of the strain to acquire DNA from the environment. This DNA was provided in two formats, plasmid or linear construct, having both a kanamycin resistance cassette, that in the case of the linear construct would recombine replacing the *pyrE* locus, a dispensable gene in rich medium. The results (Figure 4) show that, in the wild‐type background, the transformability with a plasmid is increased by more than two orders of magnitude in the RecO 1–3 series. However, for linear DNA the transformability is somewhat increased for the series, though not with statistical significance. The increase in transformation with the plasmid could likely be attributed to the fact that the *recO* mutants are also loss‐of‐function mutants in *addA* or *addB*, whose absence has been shown to increase transformation also by two or more orders of magnitude (Verdú et al., 2022). The RecR and RuvB series display an equal, or slightly higher (RecR3, RuvB1), transformability to the wild type with plasmid. At the same time, with linear DNA, the RecO mutants were slightly better than the wild type (without significance), the RecR very similar, but the RuvB series yielded no transformants. This lack of transformants in the RuvB series suggests that the RuvABC complex would be required for the resolution of intermediates that occur in the integration of linear DNA. Now, when we did this assay with the mutant series in the *ppol/addAB* background, all strains showed transformabilities typical of the *ppol/addAB* strain, which is more than three orders of magnitude higher than the wild type, no matter if using plasmidic or linear DNA. This means that the effect of the deletions of *ppol/addAB* on transformation is epistatic over the effects of the *recO*, *recR* or *ruvB* deletions.

### The recO, recR and especially the ruvB mutants show filamentation phenotypes

**FIGURE 5:**
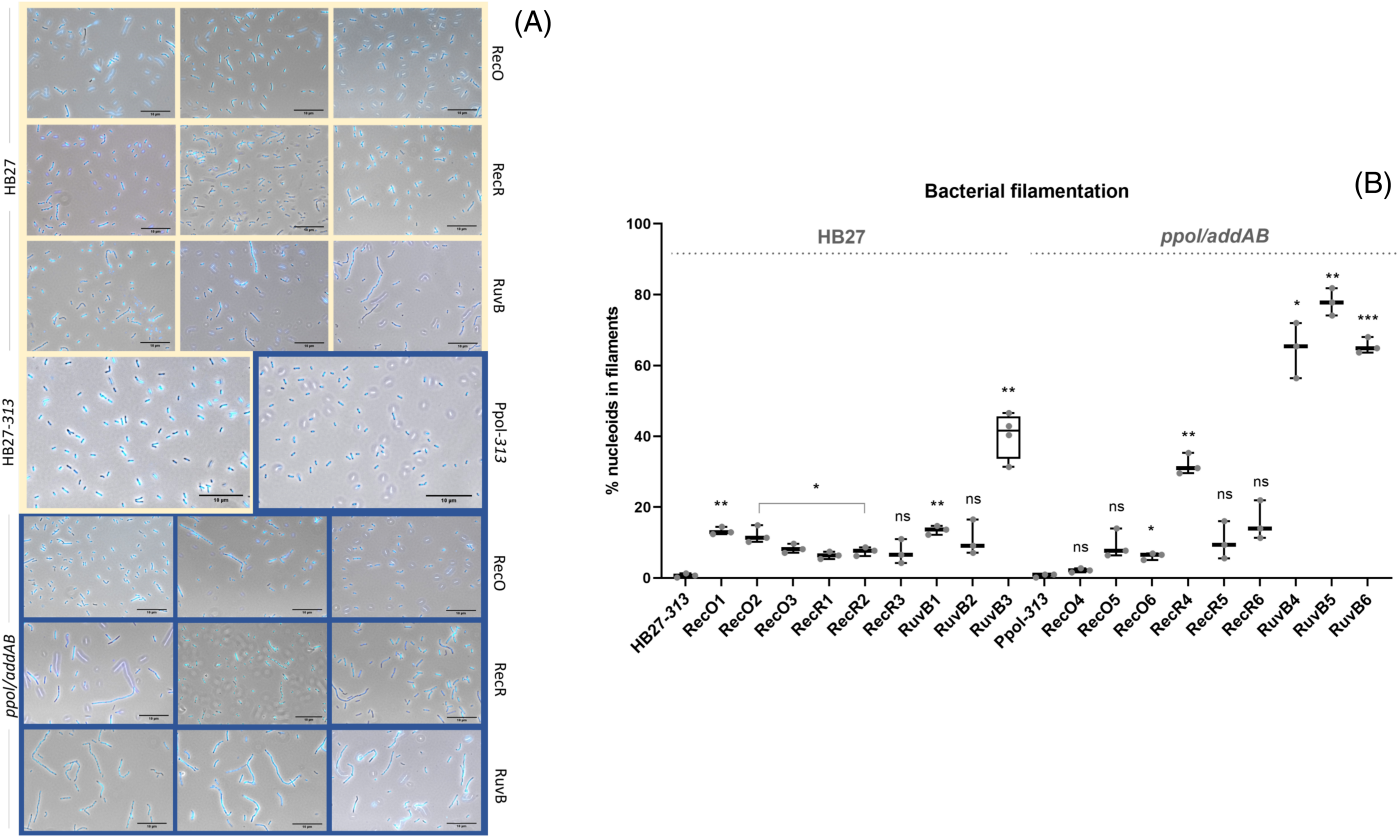
Cell division efficiency and nucleoid integrity in *Thermus thermophilus* mutants. (A) Control and mutant strains, as indicated, were observed by microscopy by over‐imposing phase contrast and DAPI staining images with ImageJ software. Left, centre and right panels in rows, correspond to representative images for mutant strains 1, 2, 3 (wild type background) or 4, 5, 6 (*ppol/addAB* background) for each gene. HB27‐313 and mutants constructed in this background are outlined in yellow, and those from *ppol/addAB* background in blue. The shape and position of the bacterial nucleoid as detected with the DAPI stain are indicative of effects on genome dynamics. Bar 10 μm. (B) Quantification of the bacterial filamentation measured by the percentage of cells with a number of nucleoids greater than 3. Asterisks indicate significant statistical differences (0.12 (ns), 0.033 (*), 0.002 (**), <0.001(***)) between the different mutant strains with the mean of control strains, either in wild type or in *pppol/addAB* background.

The different strains of this study were observed under the microscope, both by phase contrast and by DAPI nucleoid staining to detect putative defects in genome replication or segregation. Looking at the control strains HB27 and the *ppol/addAB* mutant, very few double cells or altered nucleoids were observed in overnight cultures grown at 65°C (Figure 5A). In the HB27‐*recO* mutants, a high proportion of double cells with two discernible nucleoids was apparent, but there were also found fused nucleoids specially in RecO1 and RecO2 mutants (Figure 5A). The HB27‐*recR* mutants also have some tendency to form filaments, as well as RuvB1 and RuvB2, but the nucleoids are generally condensed and well defined, while the RuvB3 mutant form longer filaments in which the nucleoids are still separated. In the *ppol/addAB* background, long filaments are apparent in the RecO5 (although not significantly) (Figure 5B), RecO6, and RecR4 mutants, with fused nucleoids in some cases. On the other hand, in the *ppol/addAB* RuvB mutant series we observed that most cells formed long filaments with RuvB4 having separate nucleoids, and RuvB5 and 6 with them commonly fused. All this suggests that in some cases the deletion of *recO* or *recR*, and generally that of *ruvB*, leads to some defects in chromosomal segregation, which in this bacterium may require recombination for chromosomal resolution. These defects are greatly enhanced in the *ppol/addAB* background for the *ruvB* mutants. Despite the evident defects in chromosomal segregation, as mentioned above, the viability does not correlate with those defects, since, for example, the RuvB mutants do not show a greatly impaired growth.

## DISCUSSION

The genus *Thermus* characteristically lives at high temperatures. At these temperatures, the DNA is under higher stress than that suffered by mesophilic organisms. Here we present a study on the proteins that form part of the RecFOR pathway, some of the most conserved factors involved in homologous recombination repair in Bacteria. Additionally, we also probed the function of the helicase RuvB, essential for the function of the RuvABC resolvasome.

**FIGURE 6:**
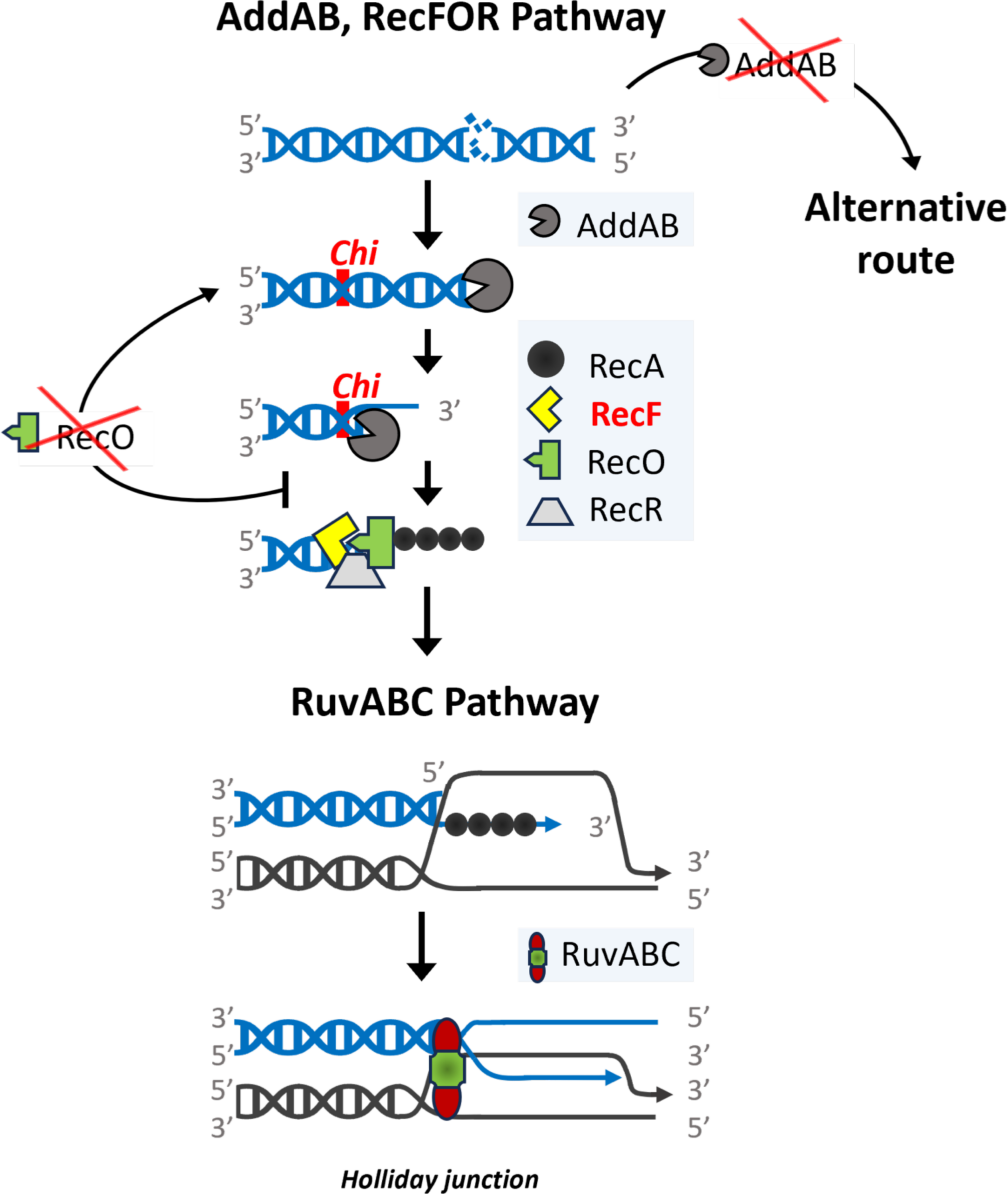
Scheme of the possible effect of deletions of recombination factors in *Thermus thermophilus*. Upon DSBs, the DNA ends would be processed preferentially by AddAB. Chi sequences would switch AddAB to produce 3′ ssDNA extensions that would allow the assembly of the RecFOR complex that in turn would charge RecA onto the ssDNA. RecA would direct the synapse that eventually could lead to Holliday junctions that would be resolved by the RuvABC complex. If RecO is missing, the DNA ends would eventually return to AddAB which would progressively generate attrition of the genome. The elimination of AddAB, on the other hand, would direct the DNA ends to an alternative route that would not generate genome attrition and would allow recombinational repair.

In our study, we sequenced the genomes of three independent clones for each one of the genes we wanted to delete, to be able to detect possible additional or compensatory changes that could have arisen during the isolation of these mutants. Our first observation was that it was not possible to construct a *recF* deletion mutant, while the *recR*, *recO* and *ruvB* mutants were easily obtained. However, all the *recO* mutants sequenced had also indels causing truncation of *addA* or *B* genes, and two of them, RecO1 and RecO3 had also point mutations in RecA at the same position, Ala80. Our explanation for the concomitant *addAB* mutations is that RecO works just after AddAB in DSB repair, displacing SSB and recruiting RecA. Thus, RecO loss of function would leave DSBs unrepaired and return the DNA ends to the very processive AddAB enzyme (Yeeles & Dillingham, 2010) that would continue the end‐degradation causing genome attrition (Figure 6). Then, upon additional loss of function of AddAB, the DNA ends would be targeted by an alternative repair route (possibly that of RecJ, also involving RecA) avoiding lethality. The relationship of the RecR and RuvB with AddAB would not be so univocal as with RecO, given that (i) we have not observed compensatory mutations in *addAB* and, (ii) the *ppol/addAB* background not only does not alleviate the defects observed in viability but aggravates those of RecR4 and 5 mutants and also the *ruvB* filamentation phenotype. In this sense, RecR would not be so critical as RecF or RecO for the RecFOR pathway, however, it could potentially play a role in other routes and, probably as a consequence, *recR* absence is aggravated by *ppol/addAB* deletion. The *ruvB* deletions have the mildest phenotype on growth, but at the same time they are the most affected in transformation with linear DNA (in the wild type), DNA damage resistance (mainly with novobiocin) and, especially, in filamentation, exacerbated in the *ppol/addAB* background. This exacerbation would not be unexpected since it has been shown that Tth Argonaute protein is involved in the decatenation of chromosomes after replication (Jolly et al., 2020) and Argonaute, in turn, can work in cooperation with RecBCD/AddAB (Olina et al., 2023). Thus, RuvB would take part in the resolution of recombination intermediates after transformation, DSBs caused by damage agents, and chromosomal decatenation, but there would also exist alternative mechanisms that would account for genome repair during normal growth conditions.

Regarding compensatory mutations, while an average of 14 mutations appears to have accumulated in the deleted strains in the wild type background, few of those could be explained based on the genes targeted or the expected phenotypes, except the ones in *recA* or *addAB*. For the *recR* and *ruvB* mutants, we could not pinpoint connected compensatory mutations, other than the upstream mutation of *recG* for RuvB1 and RuvB3.

The *ppol/addAB* mutant described in previous work appears to be genomically stable and has normal growth, but, characteristically, it has a very high transformability. In the *ppol/addAB* background, no clear compensatory mutation was apparent for any of the mutants, except for RecR6, with the change Asp138Gly in RecA that showed higher viability. The *ppol/addAB* background impairs the viability of all the mutants as well as the filamentation phenotype but not the DNA damage sensitivity. Probably, the routes in which RecR and RuvB are involved are not completely coincident with that of AddAB, so the effects add up. Also, we must point out that the *ppol/addAB* background carries a deletion in the *ppol* gene, in addition to *addAB*, that could potentially aggravate repair phenotypes. Although we have observed that the *addAB* deletion explains most of the defects in the *ppol/addAB* strain (Verdú et al., 2022), we also observed a lower plasmid stability specifically due to the *ppol* deletion. Of note, no additional *ppol* mutation was found in the HB27 RecO series, just *addA* or *addB* frameshift. The higher viability of the HB27 RecO series could be due to the presence of Ppol, or the fact that one of the AddA or B proteins would be truncated rather than being fully eliminated. Interestingly, at 70°C most of the effect of mutations on viability is abolished, which again stresses the operativity at higher temperatures of alternative route/s.

In our study, three changes in *recA* gene have appeared that could explain compensated phenotypes. In the RecO1 and RecO3 strains, in addition to AddA or B potential loss of function changes, RecA Ala80 was changed to Ser in RecO1 or to Val in RecO3. Both are conservative changes in a residue that is somewhat near the Walker A ATP binding domain, not very conserved, and for the equivalents of which there is not mutational information in bacterial *recA*s reported so far (see Figure S2), so it is difficult to predict the functional effect of such mutations (Del Val et al., 2021; McGrew & Knight, 2003; Zhou et al., 2021). In the *ppol/addAB* RecR6 mutant, with a clear improvement in viability we detected the change Asp140Gly in RecA. Asp140 forms part of the Walker B motif, although the equivalent residue in *E. coli* RecA (Figure S2) has been mutated (to Ala, Lys or Gly), and, apparently, this did not impair the protein function other than producing a constitutive coprotease activity of RecA in the case of the change to Gly (Eldin et al., 2000; Liu et al., 1993). Interestingly, Tth HB27 does not encode LexA or any similar homologous regulator (Henne et al., 2004). So, again, no predictable effect of this mutation can be suggested. However, as there is clearly a compensatory phenotype at least for *ppol/addAB* RecR6, we can hypothesize that some changes in the RecA protein would strengthen the interactions with components of alternative route/s so they can cover the absence of RecO or RecR.

When we look at the effects on DNA damage, we observed no differences with the H_2_O_2_ treatment, probably because it would activate, mostly, base‐excision repair mechanisms (Morita et al., 2010). The bleomycin treatment, on the other hand, would produce DSBs (Povirk et al., 1989), and we observe that the *ppol/addAB* background is more resistant than the parental HB27, which could be explained by the very efficient recombination occurring in the *ppol/addAB* strain (Verdú et al., 2022). The lower resistance to bleomycin of the RuvB mutant series in *ppol/addAB* is probably because the Ruv complex, as mentioned above, is also playing a role in recombinational repair in routes independent from *addAB*, so the effects of both deletions converge. The fact that in the wild type background we observe almost no differences with bleomycin treatment could be explained assuming that the AddAB activity is the predominant one in the cell. Therefore, *recR* and *ruvB* deletions would not have by themselves a detectable effect, because the AddAB pathway apparently can utilize alternative factors, starting with RecF, which could be essential. Notwithstanding, this does not preclude that RecR and RuvB play roles in other pathways and, consequently, their elimination does have effects in viability and genome repair. For the *recO* mutant the situation is equivalent to the *ppol/addAB* background, since these strains probably have an inactive AddAB complex. With novobiocin, the *ppol/addAB* RuvB series showed a significative less resistance, attributable to the defect in resolvase activity, that probably participates in the relaxation of the DNA that counters the novobiocin effect.

Regarding transformation, the HB27 RecO series show a higher transformation efficiency, both with plasmid or linear DNA, which is expected since all the clones show mutations leading to probable loss of function of AddAB. The *recR* mutants show little effect, but for the RuvB series, transformation with linear DNA is abolished, suggesting that the RuvABC resolvase complex is required in wild type HB27 for integration of DNA fragments through homologous recombination. Meanwhile, plasmid transformation would not require the resolvase and it is unchanged. The transformability levels of all the mutants are displaced on the *ppol/addAB* background, to the higher efficiencies typical of the *addAB* null background, for both plasmid and linear DNA. This again suggests that the dominant pathway would have AddAB at the start followed by RecO, RecR and, later on, RuvB. If AddAB is eliminated, the requirement for RuvB disappears, probably because an alternative route functions as backup, with RecG as putative replacement for RuvB. As mentioned, in the *ppol/addAB* mutants, alternative route/s not dependent on RecO and RecR would be efficiently performing the double recombination for plasmid or linear fragment integration, especially at higher growth temperatures. In this sense, the Tth HB27 genome codes for RecJ, RecQ, and the archaeal helicase‐nuclease pair HepA‐NurA, all of them with the potential to play key roles in recombination in *Thermus* (Blesa et al., 2017; Brüggemann & Chen, 2006; Fujii et al., 2018; Yamagata, 2001), in addition to RecF (Chaudhary et al., 2020).

Under the microscope, it is apparent that most mutants have at least some filamentation phenotype, but the defects are stronger in the *ruvB* mutants and exacerbated in the *ppol/addAB* background. The filamentation phenotype has been associated to RuvABC defects and other recombination function mutations (RecA, RecB, RecD, RecO, RecG, and others) in *E. coli* (Buljubašić et al., 2013; Ishioka et al., 1998; Zahradka et al., 1999; Zahradka et al., 2023) and in *B. subtilis* (Carrasco et al., 2004; Torres et al., 2017), commonly under DNA damage conditions, while in our case, important filamentation occurs in the absence of DNA damage. Filamentation has been detected so recurrently that it has even been proposed that it may be a post‐stress recovery mechanism (Cayron et al., 2023). Regarding the signalling leading to blocked cell division, it is worth noticing that in Tth there are no annotated genes equivalent to those of LexA, SulA, SlmA, Noc, YneA or SidA (Henne et al., 2004), which, in most studied bacteria, are key players for septum formation inhibition and, therefore, avoiding possible guillotine effect on the genome (Burby & Simmons, 2020; Wu & Errington, 2012). So, whether or not a division inhibition mechanism operates in Tth is still unknown, but is seems clear that even in the cases where more filamentation is detected, as in the *ppol/addAB* RuvB series, a proportional compromise in viability is not observed.

As mentioned in the Introduction, the RecFOR pathway, RecBCD or AddAB, and the RuvABC complexes are very conserved in Bacteria. However, in general, most of these factors are not strictly essential, because the cell has several routes with certain degree of redundancy for recombinational repair of the genome. In any case, additive negative effects have been reported for the deletion of RecBCD/AddAB plus components of the RecFOR pathway in most bacteria (Alonso et al., 1993; Buljubašić et al., 2019; Wang et al., 2011; Zuñiga‐Castillo et al., 2004). Thus, in this sense, Tth is a special case where AddAB loss of function apparently compensates the *recO* deletion and yields much higher transformability for all the mutants. Even so, it has also been reported that, in *E. coli*, a *recB* deletion suppresses the chromosomal segregation defects caused by *recO*, *ruvABC* or *recG* ablation (Zahradka et al., 2023). In *Deinococcus*, a genus evolutionarily close to Tth, *recO* and *recR* are dispensable, and interestingly, also *recF*. However, these deletions have clear negative effects on DNA damage resistance in *Deinococcus*, with *recF > recO > recR* from higher to lower effect (Bentchikou et al., 2010), and also on transformation (Ithurbide et al., 2020; Wang et al., 2019). Remarkably, as *Deinococcus* is naturally devoid of *addAB* genes (Lim et al., 2019), the RecFOR pathway would not be conditioned by the presence and activity of that complex, which probably could explain, in part, the efficient genome repair in *Deinococci*. In Tth, on the other hand, AddAB appears to be placed at the start of the preferred recombination route, it has a strong activity against incoming DNA and it is not strictly essential, but its deletion leads to compensatory mutations and genomic defects. Our results suggest that, as shown for *B. subtilis*, RecOR would be working just after, and in concert with AddAB, which would eventually be followed by the action of RuvABC for the resolution of intermediates. AddAB elimination would give way to an alternative route headed by different nuclease‐helicase enzyme/s probably with the concourse of RecF and other factors, in such a way that viability and damage resistance are only moderately affected, and transformability is highly increased. All this stresses the redundancy and derived robustness of the DNA recombinational repair mechanisms that probably underlie the ability of this extremophilic organism to live at high temperatures.

## EXPERIMENTAL PROCEDURES

### Strains and growth conditions

The strains used and isolated along this work are described in Table 1. *Escherichia coli* was grown at 37°C under stirring in liquid Luria Bertani (LB) medium or in 2% (w/v) agar plates supplemented with the corresponding antibiotics. Tth was cultured at 65°C under standard conditions, or the indicated temperatures in other cases. TB liquid medium contained trypticase 8 g/L, yeast extract 4 g/L and NaCl 3 g/L in carbonate‐rich mineral water under rotational shaking (180 rpm) or in 2% (w/v) agar plates. Kanamycin (30 μg/mL), Ampicillin (for *E. coli*) (100 μg/mL), or Hygromycin B (100 μg/mL) were used for selection as indicated.

### Plasmid construction and isolation of Δppol mutants

All the cloning and gene constructions were first amplified in *E. coli* DH5α and then transferred to Tth. The plasmids used in this work are described in Table 2. Oligonucleotides used as primers for PCR are described in Table S4. DNA manipulation and cloning were performed using standard laboratory techniques. All constructs were checked by sequencing, and Tth mutants were confirmed by PCR analysis using in all cases the primer Hyg2 (GACCGATGGCTGTGAAGAAGTAC) that hybridizes into the *hyg* cassette, and RecO7‐Dir (CTACACCATCCTGGAGATCA) for *recO*, RecR8‐Rev (CTGGTCCAGCAGGTCTT) for *recR*, and RuvB7‐Dir (AGGAGGTGGACGCGCCCGTAGAT) for *ruvB*. DNA from positive clones was sent for NGS. Genomic DNA for NGS sequencing was prepared using DNeasy UltraClean Microbial Kit (QIAGEN).

**TABLE 2:** Plasmids used in this work.

Plasmid	Description/use	Reference
pMotK1103A	Bifunctional modular vector. Kn^R^	(Verdú et al., 2019)
pyrEK	Suicide plasmid in *Tth*. Amp^R^ (*Eco*), Kn^R^	(García‐Quintans et al., 2020)
pUC18	Cloning of constructs for insertion mutants. Amp^R^	(Vieira & Messing, 1982)
pUC18 *recO::hph17*	Insertional deletion of *recO*. Hyg^R^	This work
pUC18 *recR::hph17*	Insertional deletion of *recR*. Hyg^R^	This work
pUC18 *ruvB::hph17*	Insertional deletion of *ruvB*. Hyg^R^	This work

*Thermus thermophilus* insertion knockout mutants were constructed by double recombination with a linearized DNA construct containing around 1 kbp‐long upstream and downstream regions flanking the hygromycin resistance cassette (gene *hph17*), encoding a thermostable variant of hygromycin B phosphotransferase (see Table 3). Antibiotic‐resistant clones were re‐streaked twice on selection plates to avoid the presence of wild type copies of the targeted gene since *T. thermophilus* is a polyploid bacterium to finally obtain ∆*gene::hyg* mutant. As a control for HB27 and *ppol/addAB* Hyg^R^ strains, the gene TT_C0313 was disrupted in both. TT_C0313 encodes a ferredoxin nitrite reductase and is dispensable under normal growth conditions.

**TABLE 3:** Strains used in this work.

Strain	Description	Phenotype/use	Source
*E. coli* DH5α	*supE44 ΔlacU169 (Φ80 lacZΔM15) hsdR17*, *recA1*, *endA1*, *gyrA96*, *thi‐1 relA1*	Cloning purposes	(Hanahan, 1985)
HB27	*ATCC BAA‐163/DSM7039*	Wild type	Y. Koyama
HB27‐313	Δ*TTC0313::hph17*	Hyg^R^. Chromosome labelled	(Alvarez et al., 2014)
*ppol/addAB*	Δ*ppol*, Δ*addAB*	Hypercompetent	(Verdú et al., 2022)
*ppol‐313*	Δ*ppol*, Δ*addAB* Δ*TTC0313::hph17*	Hypercompetent, Hyg^R^. Chromosome labelled	(Verdú et al., 2022)
HB27 recO(1,2,3)	Δ*recO::hph17*	Hyg^R^. Chromosome labelled	This work
HB27 recR(1, 2, 3)	Δ*recR::hph17*	Hyg^R^. Chromosome labelled	This work
HB27 ruvB(1, 2, 3)	Δ*ruvB::hph17*	Hyg^R^. Chromosome labelled	This work
*ppol/addAB* recO(4, 5, 6)	Δ*ppol*, Δ*addAB*, Δ*recO::hph17*	Hyg^R^. Chromosome labelled	This work
*ppol/addAB* recR(4, 5, 6)	Δ*ppol*, Δ*addAB*, Δ*recR::hph17*	Hyg^R^. Chromosome labelled	This work
*ppol/addAB* ruvB(4, 5, 6)	Δ*ppol*, Δ*addAB*, Δ*ruvB::hph17*	Hyg^R^. Chromosome labelled	This work
*TthHB27ppol‐pyrE*	Δ*ppol*, Δ*pyrE::kat*	Hypercompetent, Kn^R^. Chromosome labelled	This work

### NGS genome sequencing

The genomes of all the mutant strains were sequenced by the company Microbes NG (www.microbesng.com) using Illumina technology with a target coverage of 30‐fold. Reference genome for HB27 strain (NC_005835.1, NC_005838.1) was downloaded from the NCBI. The reads were aligned against the reference genome using BWA aligner (Li & Durbin, 2009). Picard Tools (https://broadinstitute.github.io/picard/) was used to clean, sort, and mark duplicates of mapped BAM (Binary Alignment Map) files. The final BAM file was used for Variant Calling (Sandmann et al., 2017). This was performed using the GATK toolkit (McKenna et al., 2010) to identify SNPs on each mutant with the HaplotypeCaller tool. The results were processed using an in‐house script written in Python language (compareSNP_betweenSamples.py) to compare all variants positions between samples and to detect the genetic location of each variant. All the sequences were uploaded to the European Nucleotide Archive (https://www.ebi.ac.uk/ena/browser/home). See Table S4 for project numbers and sample references.

The tables compiling the variants positions present in the different strains were manually curated eliminating the variants occurring in our HB27 lab stock strain respect to the NCBI reference sequence, and therefore they were also subtracted from the analysis of all the HB27 stock derived strains. Similarly, all the variants present in the initial *ppol/addAB* sequenced genome were also subtracted from the derived mutant sequences. These lists of variants of HB27, and *ppol/addAB* respect to the reference genome are shown in Tables S1 and S2, respectively, and Table S3 for the pTT27 megaplasmid.

### Transformation and DNA damage assays

*Thermus thermophilus* strains were transformed by natural competence (Koyama et al., 1986). The desired amount of DNA (100 ng or 200 ng, as indicated) was added to 0.5 mL of mid‐exponential cultures of Tth, then, after 4 h incubation at 65°C, the cells were spread on selection plates and then incubated for 2 days at 65°C or the indicated temperatures. Transformation frequencies were calculated as the number of colony‐forming units (CFU/mL) on selective plates divided by the number of CFU/mL in non‐selective plates. The frequencies were calculated by spot assays using the indicated dilutions of transformation cultures and by standard plating. The linear DNAs for transformation were generated by PCR from the plasmids pyrEK and pyrEH using M13 forward and reverse primers. A minimum of three independent experiments with technical triplicates was performed for each assay.

To study the effects of four different chemical DNA damaging agents: hydrogen peroxide (H_2_O_2_), bleomycin (Bleo), novobiocin (Nov) and 4‐nitroquinoline‐N‐oxide (4‐NQO) on the survival of Tth mutants, cultures at stationary phase were diluted to OD_600_ = 1.6 plated on TB‐agar to obtain a bacterial lawn of 10^7^ or 10^8^ cells. Then, cellulose disks (1/4′ BDC [USA]) were placed on top of TB plates without antibiotic and 10 μL of the corresponding damage agents H_2_O_2_ (0.5%), Bleo (0.1 mg/mL), Nov (0.2 mg/mL) and 4‐NQO (0.5 mM) were added to be absorbed by the disks. The plates were incubated at 65°C for 48 h and the inhibition halos were measured as the diameter in cm of the clear circle.

### Temperature effect

After 24 h of growth, the cultures were adjusted to the same optical density (OD_600_ = 1.4) and serial dilutions (1:10) were made in a sterile 96‐well plate until a final dilution equivalent to 10^−9^ was obtained. Subsequently, 10 μL of each dilution were spotted on TB‐agar plates. Plates were incubated at 60°C, 65°C and 70°C (in this case in the absence of antibiotic) for 24 or 48 h. This procedure was performed with each type of mutant, seeding the corresponding control strain on the same plate. Thermal control of the ovens used was carried out to ensure a correct temperature during the incubation of the plates with a recording device EasyLog USB Temperature and Humidity Logger (LASCAR electronics, USA). To quantify the effect of temperature on viability, colony‐forming units (CFU) per mL were calculated.

### DAPI staining and visualization

Tth strains were allowed to grow in rich media until stationary phase. Thereafter, 10^9^ cells were pelleted and washed twice with 500 μL of phosphate‐buffered saline (PBS) pH 7.4. Fifty microliters of washed cells were incubated 5 min with 0.5 μL of 4′,6‐diamidino‐2‐phenylindole (DAPI) 0.5 μg/mL stock. After incubation, cells were washed twice with 150 μL of PBS and resuspended in the same volume. Five microliters of the final cell suspension were used for fluorescence microscope visualization at 60x magnification. Images were processed with the ImageJ software (Schneider et al., 2012).

### Statistical analysis

Prior to statistical analysis, the normality and homogeneity of variance of the data obtained was verified through a Shapiro–Wilk test. In all experiments, the data obtained passed the normality test, except for the transformation efficiency data. The transformation efficiency data was log transformed to a normally distributed data before the statistical analysis explained up next. In all cases, the statistical analysis made was a One‐Way ANOVA test (Brown‐Forsythe and Welch ANOVA test), with a 95% confidence interval, and Dunnett's T3 correction for multiple comparisons for comparing the different mutant strains to the control strain in each case. All statistical analysis was performed with the R software (R Core Team, 2023).

## AUTHOR CONTRIBUTIONS

**Cristina L. Gómez‐Campo:** Data curation (equal); formal analysis (lead); investigation (equal); methodology (equal); validation (equal); writing – review and editing (equal). **Ali Abdelmoteleb:** Formal analysis (equal); investigation (equal); methodology (equal); validation (lead). **Verónica Pulido:** Data curation (equal); formal analysis (equal); investigation (equal); methodology (equal); validation (equal). **Marc Gost:** Formal analysis (equal); investigation (equal); validation (equal); writing – review and editing (equal). **Dione L. Sánchez‐Hevia:** Conceptualization (equal); investigation (equal); validation (equal). **José Berenguer:** Conceptualization (equal); funding acquisition (lead); investigation (equal); resources (lead); supervision (lead); writing – review and editing (lead). **Mario Mencía:** Conceptualization (lead); funding acquisition (lead); investigation (equal); supervision (lead); writing – original draft (lead).

## CONFLICT OF INTEREST STATEMENT

No conflict of interest to declare by any of the authors.

## Supporting information


**Figure S1.** RecF complementation assay.

**Figure S2.** Alignment of bacterial RecA sequences.

**Table S1.** List of all the mutations detected in the mutant strains generated on the HB27 wild type background with respect to the Tth HB27 reference chromosome. The position in the genome, nucleotide/s change, annotated gene and protein encoded are shown for each strain (in columns). Highlighted in orange, class 1 mutations; in yellow, class 2 present in more than one strain.

**Table S2.** List of all the mutations detected in the mutant strains generated on the *ppol/addAB* background with respect to the sequence of the *ppol/addAB* original strain chromosome. Rows and columns as in Table S1. Highlighted in orange, class 1 mutations; in yellow, class 2 present in more than one strain.

**Table S3.** List of all the mutations detected in the mutant strains generated on the HB27 wild type background with respect to the Tth HB27 megaplasmid pTT27 and on the *ppol/addAB* background with respect to the sequence of the *ppol/addAB* megaplasmid pTT27. Rows and columns as in Table S1. Highlighted in yellow, class 2 mutations present in more than one strain.

**Table S4.** List of all the genome sequence archives available in the European Nucleotide Archive for the new Tth strains reported in this article. The project and sample accesion number are given for each strain.

## Data Availability

Data available on request from the authors.
