# Supplementary material for: Differential requirement for RecFOR pathway components in Thermus thermophilus
Source: Environ Microbiol Rep. 2024 Jun 1;16(3):e13269. doi: 10.1111/1758-2229.13269 (PMC11143384; doi:10.1111/1758-2229.13269)
Supplement: Supplementary file 1 — Figure S1. RecF complementation assay. [file EMI4-16-e13269-s004.pdf]

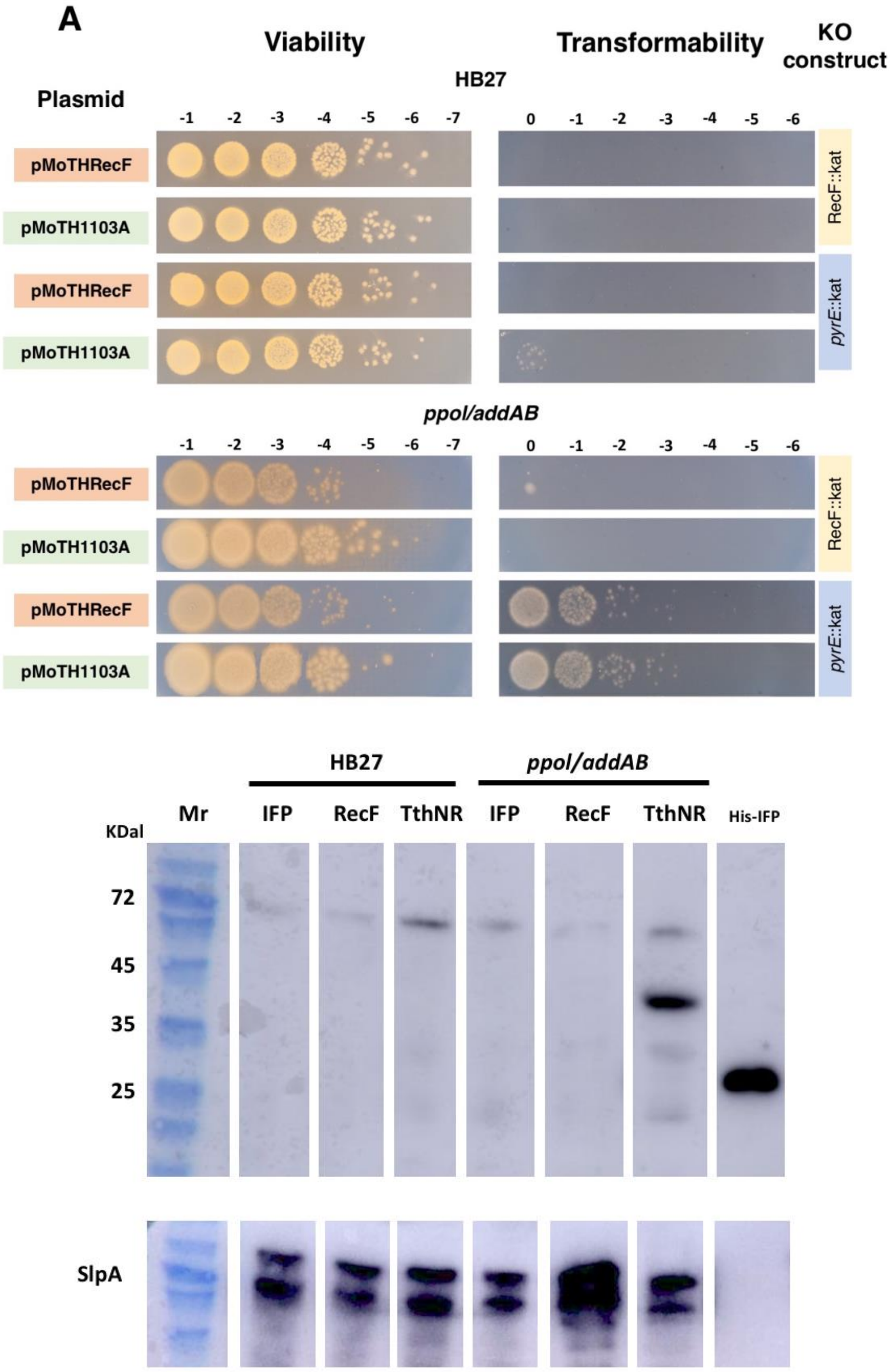
A
Viability
Transformability
KO construct
Plasmid
HB27
-1 -2 -3 -4 -5 -6 -7
0 -1 -2 -3 -4 -5 -6
pMoTHRecF
pMoTH1103A
pMoTHRecF
pMoTH1103A
RecF::kat
pyrE::kat
ppol/addAB
-1 -2 -3 -4 -5 -6 -7
0 -1 -2 -3 -4 -5 -6
pMoTHRecF
pMoTH1103A
pMoTHRecF
pMoTH1103A
RecF::kat
pyrE::kat
HB27
ppol/addAB
Mr
IFP
RecF
TthNR
IFP
RecF
TthNR
His-IFP
KDal
72
45
35
25
SlpA

Supplementary Figure 1. RecF complementation assay. A) Serial dilutions of cells (Tth HB27, or *ppol/addAB* strains) harboring the indicated plasmids (pMoTHRecF, expressing His-tagged RecF, or pMoTH1103A expressing IFP, both conferring resistance to hygromycin) were transformed with the constructs to generate the indicated deletions, by substitution with the kanamycin resistance cassette. Cells were subsequently spotted in plates supplemented with hygromycin (left pannels, Viability), or in plates supplemented with hygromycin and kanamycin (right pannels, Transformability) and incubated at 65°C for 48 hours. **B)** Western blot detection of His-tagged proteins expressed from the corresponding plasmids from HB27 or *ppol/addAB* cultures. Mr, Molecular weight standard; IFP, Citrine version of GFP expressed from pMoTH1103A; RecF, vector to express His-tagged RecF, pMoTHRecF; TthNR, His-tagged non-relevant Tth protein expressed from pMoTH; His-IFP, purified His-tagged IFP protein (50 ng); SlpA panel, loading control of the same membrane probed with anti-SlpA (S-Layer) protein antibody (monoclonal 1AE1) (Olabarria et al., 1996). MWs, IFP, 26,7 KDal; RecF, 37,8 KDal; TthNR, 72,9 KDal. The TthNR band mobility in strain *ppol/addAB* could be due to proteolysis.

Supplementary method

Western Blotting. Tth cells transformed with the corresponding plasmids were cultured overnight, and then total cell extracts were prepared by resuspending 10^9 cells in 200 µL of loading buffer and boiling for 10 min. The extracts were analyzed by SDS-PAGE, electrotransferred to a PVDF membrane, and detected with specific antisera (HisProbe^{TM}-HRP) (Thermo Scientific), and, for loading normalization, anti-SlpA (S-layer protein monoclonal antibody). The membranes were developed using horseradish peroxidase-labeled antibodies and bioluminescence assay (ECL, Amersham International). Western blot band detection was performed with an Amersham Imager 680 chemiluminescence reader.

Reference

Olabarria, G., Carrascosa, J.L., de Pedro, M.A. & Berenguer, J. (1996). A conserved motif in S-layer proteins is involved in peptidoglycan binding in *Thermus thermophilus*. *Journal*

of Bacteriology 178, 4765–72. Available at: https://doi.org/10.1128/jb.178.16.4765-4772.1996
