# Supplementary material for: Differential requirement for RecFOR pathway components in Thermus thermophilus
Source: Environ Microbiol Rep. 2024 Jun 1;16(3):e13269. doi: 10.1111/1758-2229.13269 (PMC11143384; doi:10.1111/1758-2229.13269)
Supplement: Supplementary file 2 — Figure S2. Alignment of bacterial RecA sequences. [file EMI4-16-e13269-s003.pdf]

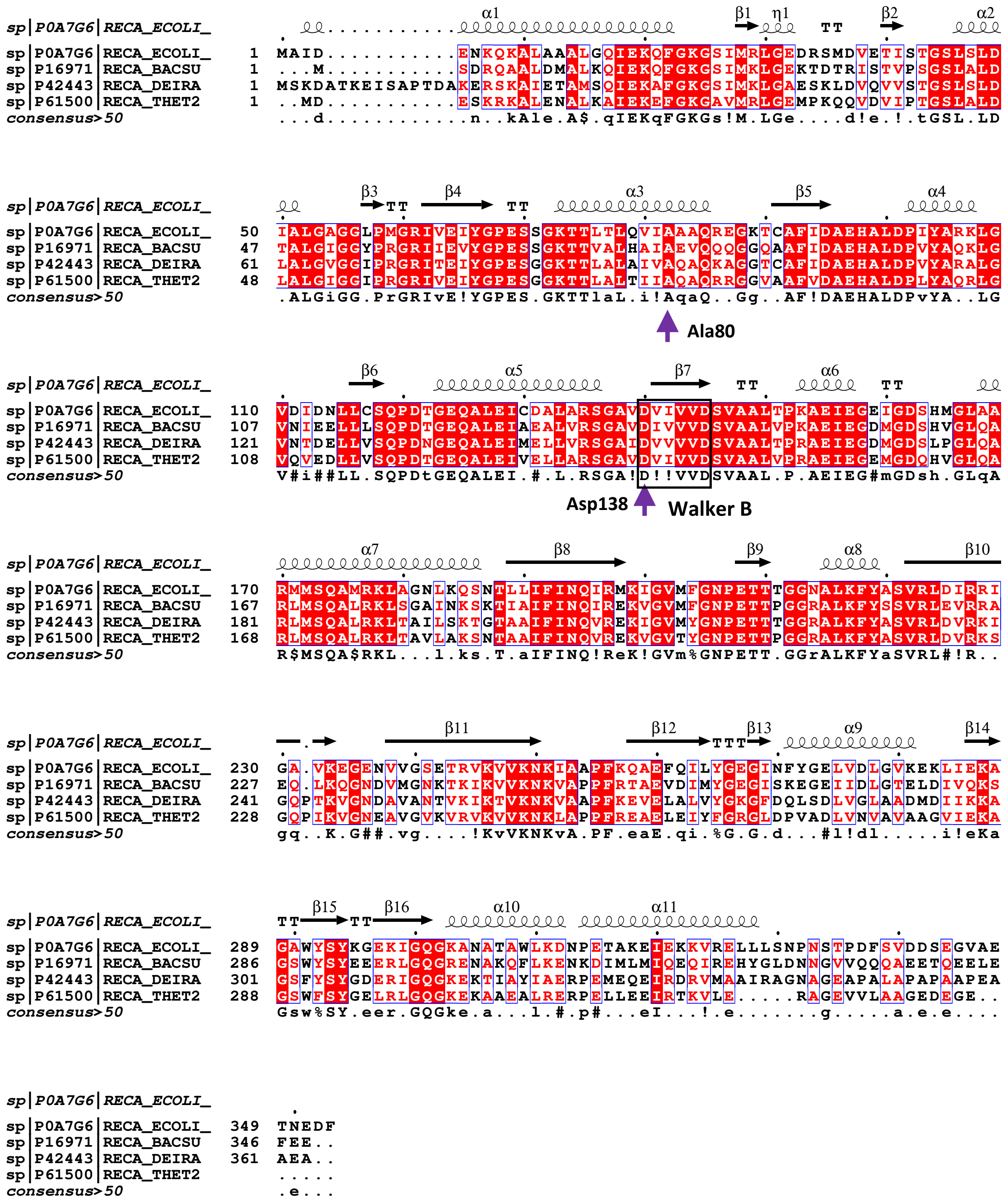


**Supplementary Figure 2.** Alignment of bacterial RecA sequences. The RecA protein sequences of *Escherichia coli* (ECOLI), *Bacilus subtilis* (BACSU), *Deinococcus radiodurans* (DEIRA) and *Thermus thermophilus* (THET2) (with the corresponding Uniprot entries as indicated) were aligned using the software MUSCLE (https://www.ebi.ac.uk/Tools/msa/muscle/) and represented using the ESPript 3.0 server https://espript.ibcp.fr/ESPript/ESPript/ (Robert and Gouet, 2014). Changes in the Tth RecA are indicated with purple arrows.

## Reference

Robert, X., Gouet, P., 2014. Deciphering key features in protein structures with the new ENDscript server. Nucleic Acids Research 42, W320–W324. https://doi.org/10.1093/nar/gku316
